# Edge Computing Deployment Algorithm and Sports Training Data Mining Based on Software Defined Network

**DOI:** 10.1155/2022/8056360

**Published:** 2022-05-27

**Authors:** Minggang Yang, Cuifang Gao, Junmei Han

**Affiliations:** ^1^School of Physical Education, Shandong University of Technology, Zibo, Shandong 255000, China; ^2^Department of Traditional Chinese Medicine, Shandong Drug and Food Vocational College, Zibo, Shandong 255011, China

## Abstract

The wireless sensor network collects data from various areas through specific network nodes and uploads it to the decision-making layer for analysis and processing. Therefore, it has become a perception network of the Internet of Things and has made great achievements in monitoring and prevention at this stage. At this stage, the main problem is the motive power of sensor nodes, so the energy storage and transmission of wireless sensor network is imminent. Mobile edge computing technology provides a new type of technology for today's edge networks, enabling it to process resource-intensive data blocks and feedback to managers in time. It is a new starting point for cloud computing services, compared to traditional cloud computing services. The transmission speed is more efficient and will be widely used in various industries and serve them in the future. Among them, education and related industries urgently need in-depth information, which in turn promotes the rapid development of data mining by sensor networks. This article focuses on data mining technology, mainly expounds the meaning and main mining methods of data mining technology, and conducts data mining on sports training requirements from the aspects of demand collection and analysis, algorithm design and optimization, demand results and realization, etc. Monitor the training status and give the trainer reasonable suggestions. Through the processing of the training data mining results and proofreading the database standardized training data, we can formulate a personalized program suitable for sportsmen, reduce sports injuries caused by no trainer's guidance, and open new doors for training modes. Therefore, this paper studies the sensor network technology, edge computing deployment algorithm, and sports training data mining.

## 1. Introduction

The most common distributed network is the wireless sensor network, which is composed of sensors, microcontrollers, communication modules, and energy storage modules that can collect information about the surrounding environment. Each unit uses a wireless network for transmission and finally collects the information in the monitoring area [1]. The received information is submitted to the manager. Wireless sensor networks are mainly used in environmental monitoring, disaster prevention, medical care, border defense, and other fields [2]. However, wireless sensors still have security threats such as the security and stability of network transmission, fragility, difficulty in deployment, and subsequent maintenance costs [3]. Among them, it is extremely obvious that the motive force is difficult to maintain the stable operation of the sensor for a long time [4]. Because the energy of each sensor is independent but the energy storage is small and the energy exchange is difficult due to the environment, the power level directly affects the network operation time, and each unit will stop working due to the existence of contact, which will cause the entire network to fail to operate [5]. Therefore, the transmission and storage of the motive force are particularly important, which is also the foothold of the wireless sensor network. The original intention of this article is also the same. It conducts in-depth research on wireless sensor networks and makes some guidance and suggestions in theory and reality. Mobile edge computing technology provides mobile users with IT and cloud computing platforms [6]. Through wireless networks, its fast speed and large bandwidth provide users with short-distance deployment anytime and anywhere, making it possible for some computing-intensive applications to personalize services [7], thereby reducing operating costs. In recent years, many documents have focused on and explored how to provide users with highly resource-intensive services anytime and anywhere and achieved remarkable results through mobile edge computing technology [8]. Data mining technology has been deeply applied in the field of sports events, especially in foreign countries. One of the most notable is the advanced system applied in the NBA, which can analyze the historical performance of the players to obtain the players' shooting hot spots, offensive and defensive tendencies, and match-up performance, which can assist the coach to better judge the court [9]. The situation thus makes adjustments to players and tactics. In the NFL, Schatz used statistical analysis of the historical data of each team to get: the relationship between the score value and the success rate of a specific player's offensive tactics. In the NCAA, through data mining of historical records between various colleges and universities, the annual championship of each event is predicted, and the average accuracy rate can reach 84.5%. In the NHL, in order to enable people to see the data of the game anytime and anywhere, the real-time data recording and analysis system is used for the first time, so that the data of the game can be transmitted to the coach, fans, and the media in real time. In the field of volleyball, you can understand the opponent's historical record and the recent performance of each player before the game. During the game, assist the head coach to judge the situation on the field and make analysis and decision-making. After the game, the players will be trained according to the data. In the sports field, data mining and other computing techniques have been widely used. The database established by the various data of each athlete helps athletes improve their own strength more scientifically through integrated analysis and at the same time provides more scientific and effective rationalized data for coaches and other sports-related departments [10].

## 2. Related Work

In response to the above problems, the literature has developed a real-time monitoring of sports training quality and a temporary tactical adjustment system, and it conducts comprehensive and three-dimensional monitoring of athletes and records training and competition data, and then analyzes and organizes them to construct an accurate data covering all aspects of athletes [11]. The database is more accurate and comprehensive than manual records. Data mining technology is used to conduct in-depth analysis of the database, so as to formulate an athlete's ability map, and objectively and scientifically evaluate the athletes, so that the value of the data can be fully reflected. Based on the ability map, the results are applied to the daily training of each athlete, so as to improve the training plan to make up for the shortcomings and strengthen the strengths. At the same time, the coach can have a clearer understanding of each athlete's ability to assign tasks and avoid the shortcomings of the past. The literature believes that the responsibilities of the MAC layer protocol and the network layer protocol are different, but the service objects are all cross-layer protocol models, which are the allocation method of the correction channel, its error control method, and the correct path selection method [12]. And in some special cases, the simulation verification of the average energy consumption, data transmission volume, and network life cycle of the EOCP protocol has been completed compared with the comparison of the four protocols of EAUCF, DEEC, TEEN, and LEACH [13]. The literature indicates that the training methods used by the athletes at this stage are still formulated according to the coach's subjective experience and the athlete's physical condition, and there is a lack of scientific and effective data as a reference. Therefore, the coach's focus is still biased towards the training of hard physical indicators, such as endurance, speed, and strength, but lacks the training of athletes' main abilities and their specific inclination, as well as their responsibility in specific tactics, and it cannot scientifically, objectively, and systematically deal with athletes. Therefore, after data collection, statistics, analysis, and processing have obtained the athlete's personalized data, it is important for the athlete to develop a specialization plan. The literature calls the time of request response in the framework of mobile edge computing and the time of user data to the edge server in the area as communication delay [14], when the time delay is processed on the edge server and when the bandwidth capacity of the edge network is less than the requested amount. In the case of exploring the impact of VCR deployment on the request and response delay, the wireless communication delay of the edge server and its nearby mobile users is often regarded as an invariant. Because the location of the VCR has a small impact on the request response delay of the edge server and nearby mobile users, it has a great impact on the data routing in its network and the delay caused by the data processing in the edge network. When a wide variety and a large number of VCRs are deployed in a large-scale edge processor, the time delay caused by data routing in the network and data processing in the edge network is unimaginable. Therefore, in order to solve the problem of high latency of request response, VCR must be optimized and deployed. The literature believes that the application field of the WSN network is extremely wide, so the in-depth study of the WSN network has very important practical significance. Traditional cellular communication networks need to build fixed communication base stations, and they need to be evenly distributed, but the construction speed is slow, the construction and subsequent maintenance costs are high, the scalability is poor, and they are easily attacked by the network layer and cause paralysis [15]. In comparison, WSN networks are much better and can be built in extremely harsh environments, especially in the military field.

## 3. Basic Exploration of Sensor Network and Edge Computing Deployment Algorithm Research

### 3.1. Overview of Wireless Sensor Networks

**Figure 1:**
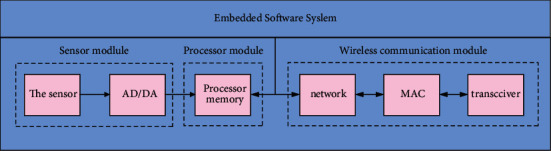
Sensor node structure.

The sensor node is generally composed of five parts, and there are 4 parts in Figure 1. However, the power supply part is not marked, because the other four parts are powered by the power supply module.

Because the sensor network needs to place many sensor nodes, the sensor must be low in price and small in size. The size of the battery directly affects the energy storage of the battery, which in turn affects the operation of the sensor node. At the same time, the detection environment of the sensor is too harsh, coupled with the requirement of low cost, and it is necessary to castrate part of the performance to improve its toughness, such as the small change in the performance of the battery affected by temperature, so the energy storage value of the battery cannot meet the sensor long-term stable operation in harsh environments. In addition, due to the large number of sensors and the harsh environment, it is impossible to replace the battery of a certain node. Even if it can be replaced, then the operating cost will inevitably increase, contrary to the original intention.

**Figure 2:**
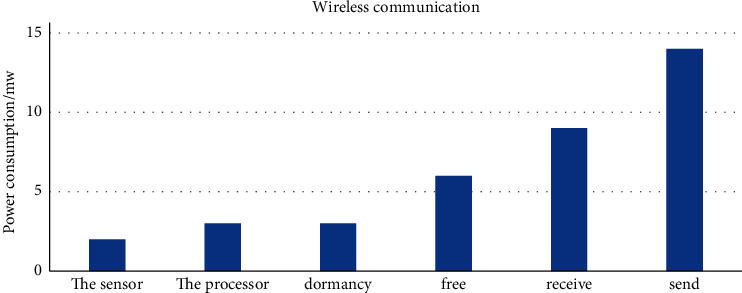
Schematic diagram of energy consumption ratio.

The data collection, transmission, and processing power consumption of sensor nodes are relatively high, so the battery equipped in sensor nodes is not enough to maintain its long-term stable operation. Even if the rapid growth of modern process technology makes the power consumption in data acquisition and processing continues to decrease, the power consumption in data transmission is still far greater than the other two aspects. A schematic diagram of the ratio of energy consumption is shown in Figure 2.

**Figure 3:**
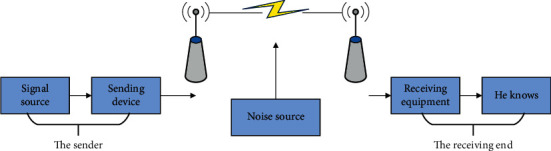
Wireless communication system model.

Electromagnetic waves propagate in free space, which is the essence of wireless communication. The traditional wireless communication system is divided into two parts: the sending end and the receiving end. The sending end consists of a signal source and a sending device, and the receiving end has a receiving device and a sink, as shown in Figure 3.

For the sensor node, it has both the sending end and the receiving end. Therefore, sensor nodes can not only send signals but also receive signals. Therefore, this article needs to have a scientific and accurate understanding of the sending device and the receiving device, so that the power consumption of the sensor node can be accurately and scientifically obtained.

As a kind of Internet of Things, it is also a very special kind. The wireless sensor network is vividly compared to the “eyes” of the Internet of Things. According to the meaning of the Internet of Things, the meaning of wireless sensor network can be obtained: in order to analyze, manipulate, judge, and manage things in the real environment, the entire wireless sensor network system is composed of countless and diverse sensor nodes. Therefore, sensor nodes are the top priority of WSN.

Through the above description, the wireless sensor network and the Internet of Things are not exactly the same. The comparison results are shown in Table 1. Therefore, the wireless sensor network and the Internet of Things are compared in terms of definition, usage, information source, basic network, and perception objects. It is obvious that the wireless sensor network is a simplified version of it.

Of course, the way of researching problems is always starting from the simplest place. Therefore, it is very correct to explore the wireless sensor network model and its optimization algorithm, which can promote other network forms of the Internet of Things to a certain extent.

The number of sensor nodes in the detection area of the sensor network directly determines the communication capability of the sensor. Therefore, the energy loss and communication distance are directly related to the damage to the communication capability, so a simple expression can be drawn:

(1) $$\begin{array}{c} E=kd^{\prime \prime }. \end{array}$$

Therefore, the power consumption of the transceiver during operation is the sum of the power consumption of the transmitter and receiver:

(2) $$\begin{array}{c} P_{C}=P_{O}+P_{TX}+P_{RX}. \end{array}$$

When designing the MAC layer protocol, nodes cannot be closed and opened frequently. Frequent opening and closing causes a certain amount of energy loss. During data transmission, all data are synchronized to the same time and transmitted together, so that the power consumption during transmission can be greatly reduced. However, all nodes are in the off state during transmission, but this is only an ideal state. Therefore, the turn-on power consumption ST is

(3) $$\begin{array}{c} E_{ST}=p_{LO}.T_{ST}. \end{array}$$

The sensor node not only loses energy in the process of opening and closing but also loses energy in the conversion from the receiving end to the transmitting end. The power consumption *w* is defined as

(4) $$\begin{array}{c} E_{sw}=P_{LO}\cdot t_{sw}. \end{array}$$

IFA is an intermediate frequency amplifier, VFD is a multifrequency mixer, LNA is a low noise amplifier, and DM is a demodulation module. The power consumption of Fs and VCO can be expressed as *n*, and the received power consumption is

(5) $$\begin{array}{c} E_{RX}=\left(P_{LO}+P_{RX}\right)t_{RX}. \end{array}$$

When data are transmitted, modules such as FS, VFD, IFA, and DM are low noise amplifier, multifrequency mixer, intermediate frequency amplifier, demodulation module, etc. The power consumption of the transmitting end in an ideal state is

(6) $$\begin{array}{c} E_{TX}=\left(P_{L}+P_{PA}\right)t_{Tx}. \end{array}$$

Because the RF power increases, the power consumption of the amplifier increases, so the total power consumption increases. Therefore,

(7) $$\begin{array}{c} P_{PA}=\frac{1}{\eta }P_{out}. \end{array}$$

Because the transmit power of RF is affected by the distance *d*, the expression for the distance *d* and the power consumption of the power amplifier is

(8) $$\begin{array}{c} P_{PA}=\frac{1}{\eta }\gamma_{PA}\cdot r\cdot d^{n}. \end{array}$$

According to the above expression, it can be concluded that the total energy consumed by the node is the sum of the energy consumed by each part:

(9) $$\begin{array}{c} E_{C}=E_{sT}+E_{RX}+E_{sw}+E_{TX}. \end{array}$$

Assuming that the transmission and reception duration can be expressed as *K*=*K*=*K*=*∗T*, where PKT is the length of the data packet. Using (10), the total power consumption can be obtained as

(10) $$\begin{array}{c} E_{C}=P_{LO}\left(t_{sT}+t_{sw}\right)+\left(2P_{LO}+P_{RX}\right)\frac{l_{PKT}}{r}+\frac{1}{\eta }\cdot \gamma_{PA}\cdot d^{n}\cdot l_{PKT}. \end{array}$$

### 3.2. Research on Edge Computing Deployment Algorithm

**Figure 4:**
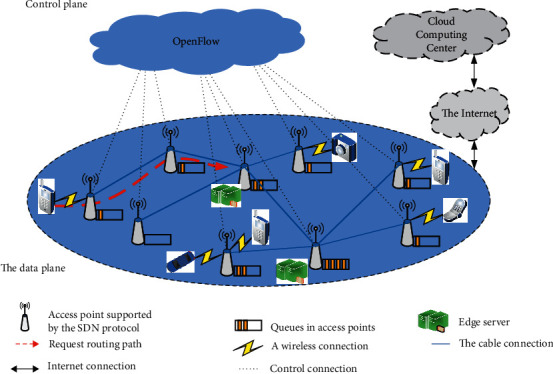
Edge network architecture based on SDN technology.

This article focuses on the way the edge server accepts service requests from mobile users, which is mainly transmitted through the AP route in the service model of the edge network, and the main basis is SDN technology. Therefore, a large number of APs need to be placed to support a large number of devices that can connect to the edge network and obtain services at any time during the movement. The quality of service when requesting data is transmitted from the AP to the edge server depends on the transmission delay. However, the transmission delay of data between the AP and the edge server is affected by the total bandwidth of each switch through which the data passes. When high latency occurs in the edge network, the main reason is that the bandwidth of the data transmitted on the switch through which the data are transmitted is too small, so as shown in Figure 4, how to better place the edge server in the space of the edge network, making the edge server respond faster to mobile user requests is a question worth thinking about.

**Figure 5:**
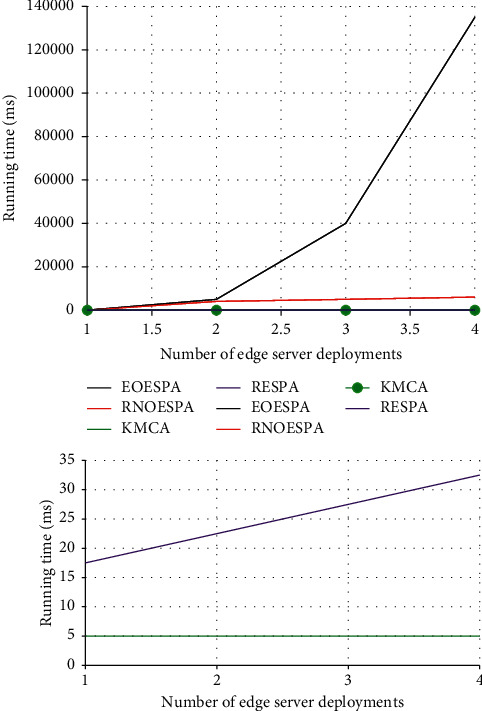
Computational complexity comparison of algorithms.

It can be seen from Figure 5 that the number of edge servers placed in the edge network directly affects the speed at which the edge server responds to the request of the user to initiate it. One thing in common among the four different solutions is that when the number of edge servers increases, the delay in responding to requests will be significantly reduced.

**Figure 6:**
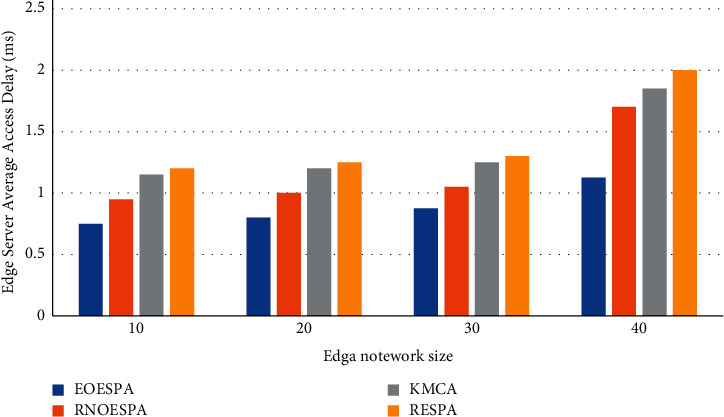
The influence of the number of edge servers on the average access delay of edge servers.

Figure 5 compares the computational complexity of these algorithms. It can be seen from Figure 6 that the time complexity of these four schemes will all affect their complexity due to the increase in the number of edge servers placed. Among them, EOESPA is the most affected.

**Figure 7:**
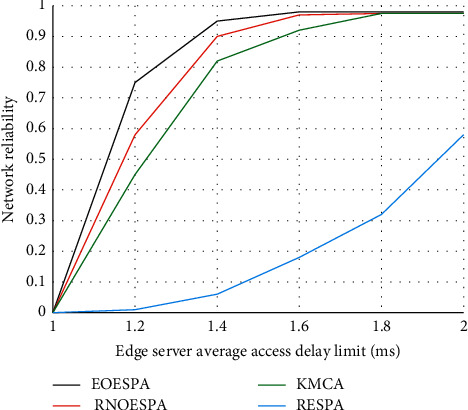
Comparison of network reliability under the constraints of edge server access delay.

In the end, EOESPA has obvious advantages over the other three options.  Figure 7 shows that the parts in each server node cannot work normally due to aging, which affects the stability of the edge server when working, and then causes the node and even the entire line to be paralyzed. Faced with this situation, the performance of RNOESPA is compared with other Three options are better.

KMCA calculates the average delay generated by the edge server at the end of an iteration and judges whether it converges. When it converges, it proves that the optimal solution has been found. Otherwise, the next calculation will be performed until it converges. *O* is the computational complexity of KMCA. *T* is the maximum number of iterations. The comparison of the computational complexity of the four algorithms is shown in Table 2.

In order to scientifically and accurately reflect the transmission delay of the edge server in response to its service request, this paper uses the Python programming language to build a model of the edge server responding to the service request, which is mainly based on the edge network structure of the SDN technology, so as to calculate the performance of the proposed algorithm in this paper more accurately. In this paper, ∈=2 is used to construct a real edge network topology structure with a simulation topology, which is mainly based on the random network generation method. As described in detail in Table 3, due to the high cost of building edge servers, the deployment nodes are far from reaching the scale of SDN access points. Therefore, the value of edge server in this paper is [1, 4] and the number of APs is [10,40] to simulate the relationship between the two in the real environment.

If more than 50% of the mobile users' requests within the deployed server range are responded by the server through the edge network, the average distance between the device and the server can be obtained, and then the smallest average distance can be compared using GSDA and a VRC can be deployed and then arrive at the optimal plan. The comparison between the computational complexities of these several algorithms is shown in Table 4.

In addition, it can be seen from Table 5 that due to the increase in mobile users and their service requests, the delay has also increased, especially under the SEHSDA scheme, where their growth rates are almost the same.

The AP arrives at the edge server via APi and is used to receive requests from mobile users in the area. So, the service request response rate of APi can be described by the following formula:

(11) $$\begin{array}{c} \lambda_{i}=\sum_{n,meV}\sum_{f\in C}I_{m,f}\cdot \delta_{j,n}\cdot P_{n,m}^{i}\cdot R_{n},\forall i\in V. \end{array}$$

The process of service request data waiting to be transmitted on the AP is just like the M/M/1 queue. The variable *μi* is used to represent the data transmission rate of A. In the model proposed in this chapter, the data transmission rate *μi* is determined by the data transmission capacity of A and the size of the service request data volume is determined:

(12) $$\begin{array}{c} \min\frac{\sum_{i\in V}\lambda_{i}/-\left(\mu_{i}-\gamma_{i}\right)}{\sum_{i\in V}R_{i}}. \end{array}$$

Assuming that all APs will receive all sent data packets, based on this assumption, *λi*/(*μi* − *λi*) can be used to evaluate the number of service requests waiting to be transmitted in the service queue in A:

(13) $$\begin{array}{c} \sum_{j\in C}I_{i,f}=1,\forall i\in V. \end{array}$$

On the whole, the AP in the area where the mobile user is located transmits all of its user's service requests to the edge network server, and after the server responds and processes it, it exits the system:

(14) $$\begin{array}{c} \sum_{i\in V}I_{i,j}=1,\forall j\in C. \end{array}$$

In the edge network based on SDN technology, the minimum average time required for mobile devices to send service requests to the edge server is reduced in the best way, and the response and processing of the edge server are obtained, that is, the deployment of the server is optimized:

(15) $$\begin{array}{c} \mu_{i}>\lambda_{i},\forall i\in V. \end{array}$$

Intermediate centrality is a commonly used indicator for centrality measurement. It refers to the ratio of the shortest path passing through A and connecting other two APs to the total number of shortest paths between these two APs in the edge network. It is defined as follows:

(16) $$\begin{array}{c} \beta_{i}^{b}=\sum_{s,t\in V}\sum_{j\in C}\frac{l_{t,j}\cdot \delta_{js}\cdot P_{s,t}^{i}}{I_{t,j}\cdot \delta_{j,s}},\forall i\in V. \end{array}$$

The load centrality of *A* refers to the proportion of paths passing through *A* among all routing paths in the edge network, which is defined as follows:

(17) $$\begin{array}{c} \beta_{i}^{l}=\frac{\sum_{s,t\in V}\sum_{j\in C}I_{t,j}\cdot \delta_{js}\cdot P_{s,t}^{i}}{\sum_{s,t\in V}\sum_{f\in c}I_{t,j}\cdot \delta_{js}},\forall i\in V. \end{array}$$

According to the information entropy theory, to reduce the information entropy of a link attribute, it is necessary to increase its dispersion, but the moderate impact on its AP increases. Therefore, the information entropy of the connection attribute is obtained by the normalization of its AP. The calculation process is as follows:

(18) $$\begin{array}{c} H_{\beta }=\frac{-\sum_{t\in V}\beta_{l}\ln\;\beta_{i}}{\ln\left|V\right|}. \end{array}$$

When the connection attribute has a higher entropy value, it means that the connection attribute of all APs can provide less information. The information utilization rate of each connection attribute is based on the difference between its entropy value and 1. Based on the entropy value of each connection attribute, the weight of the entropy of each connection attribute is defined as

(19) $$\begin{array}{c} \omega_{\beta }=\frac{1-H_{\beta }}{\left|A\right|-\sum_{\beta \in A}H_{\beta }\;}. \end{array}$$

The sum of the weights of all connection attributes on the normalized impact factor of the deployed edge server is also used as a dependent variable. The appropriateness of each AP as an edge server placement location is calculated as follows:

(20) $$\begin{array}{c} W_{t}=F_{t}\cdot \lambda_{i}\\ =\sum_{\beta \in A}\omega_{\beta }\beta_{i},\forall i\in V. \end{array}$$

The overall network delay incurred when the edge server *u* requests to provide application *m* services for mobile devices within the coverage area of the edge server *v* is

(21) $$\begin{array}{c} {\text{T}}_{v,u}^{m}=\delta_{v,u}^{m}\cdot R_{v}^{m}\cdot \sum_{e_{i}\in E}D_{e_{i}}^{m}P_{v,u!}^{e_{l}}. \end{array}$$

For different types of application services, the request data for resource requirements are also diversified. The variable *D* is used to represent the average transmission delay of the request data of the application service *m* on the link *ei*:

(22) $$\begin{array}{c} 0\leq \delta_{v,u}^{m}\leq 1,\forall v,u\in V,\forall m\in M. \end{array}$$

The network delay when the service request processing result is sent back to the transferring device. The different configurations of VRC supporting different application services in the edge network will not affect the one-hop transmission delay between the mobile device and the nearby edge server:

(23) $$\begin{array}{c} 0\leq \alpha_{v}^{m}\leq 1,\forall v,u\in V,\forall m\in M. \end{array}$$

The process of each edge server providing services for application service requests can be regarded as an M/M/1 queuing model:

(24) $$\begin{array}{c} {\text{T}}_{v}^{s}=\frac{1}{\mu_{v}-\sum_{m\in M}A_{v}^{m}}. \end{array}$$

In summary, the average processing delay of the edge server in response to the service request is

(25) $$\begin{array}{c} \sum_{u\in V}n_{u}=N,\forall n_{u}\in U. \end{array}$$

## 4. Research and Analysis of Sports Training Data Mining

### 4.1. Classification of Sports Training Data Mining

A two-dimensional table is composed of rows and columns. Multiple two-dimensional tables constitute a relational database. At the same time, the relationship between rows and columns and two-dimensional tables is also represented by the relational database, and the entire relational database is user-oriented. Because of its special relationship, users can use their own prison to query the information they want, and display-related information along with the information. Because the database is very common and easy to operate, it is often used for data mining. Data warehouse transports, stores, and organizes data from different types and regions to make it organized and facilitate subsequent data mining. Therefore, data warehouses are widely used in all walks of life and are a very common data storage library. Especially, in the field of affairs, because of its easy operation, large storage capacity, and high security, data are often stored in a database, such as banking and accounting. Because of these fields, you can often use a special number string to represent the entire event. For example, banks can number customers and add suffixes to them, such as the deposit date and amount, as well as the balance and withdrawal location, and so on. The time database for storing time-related information, the spatial database for storing space-related information, and the temporal database together constitute a spatiotemporal database. For example, personal information updated over time, weather information, geographic location information, news data, ebb and flow of tides, sunrise and sunset, etc. For example, the music you hear and the video you see every day can be stored in the multimedia database in the form of a file after a special binary encoding and can be decoded and searched through the special decoding method of the server. The textual description of any document can be stored in the text database. At the same time, the text database can also be said to be the largest information database.

**Figure 8:**
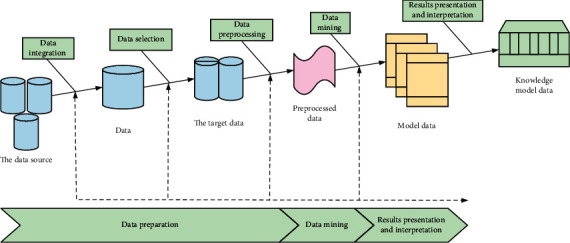
Data mining step diagram.

The steps of data mining are generally divided into: data preparation, data mining, result presentation, and interpretation. As shown in Figure 8.

### 4.2. Demand Analysis of Training Quality Monitoring and On-the-Spot Tactics Statistics System

Compared with the past, coaches can now see the dynamic quality of athletes' training and their physical state and performance on the court anytime and anywhere and collect athletes' historical training data and competition data to pave the way for later data mining.

In previous competitions, coaches' data collection often relied on their own subjective judgments and actual experience, and the collection area could only cover athletes' daily training and competitions to evaluate the athlete's training quality and competition status. However, for a long period of time, a large number of discrete data cannot be correctly counted which makes coaches unable to scientifically and objectively make accurate judgments of athletes' abilities. At the same time, the data cannot be stored for a long time, and it is inconvenient to carry, making it impossible to maximize the effect.

The use of mobile terminals as information collection and use tools, training quality monitoring, and on-the-spot tactical statistical system design can better solve the above problems. It is easy to carry and can be stored for a long time, and the collected data can also be completely and comprehensively stored. In order to obtain more scientific and accurate statistical results and to rationalize the evaluation of athletes, data mining technology is used to conduct in-depth mining of the athlete's database. Provide reliable data support for the formulation of the coach's training plan, the monitoring of training quality, and the adjustment of on-the-spot tactics.

In order to clearly obtain the data transmission in the whole system network, as well as its external data, mobile users and their application-side servers, and servers and their application-side and network system databases. This article uses data transmission analysis technology to focus on the research on the data transmission direction of mobile users and their databases. The mobile functional end can be used as the data output end to display the data results and can also upload the streamed data to the server functional end for data deepening processing; the server functional end can receive data input from external documents and can also analyze the results of the data Output, and the data can be stored in the database.

### 4.3. System Design of Training Quality Monitoring and On-the-Spot Tactics Statistics System

In the wireless network environment, the coaching staff can use the network terminal to understand the training situation and competition status of each athlete in real time. You can also use network technology to achieve comprehensive utilization of database resources anytime and anywhere. All collected data will be uploaded to the server, which stores the training plans and results of a large number of coaches and athletes. At the same time, to ensure the security of the data, a firewall is set up in the front of the server. Athletes build a system structure according to their training needs.

The system mainly includes two main parts, the mobile terminal and the server terminal, each of which contains five application modules. According to the athlete's training situation and on-the-spot performance, the mobile terminal can be used to directly enter relevant data information, and you can immediately understand the athlete's training level and the score during the game, the tactical performance, the rate of receiving and serving scores, and other important indicators. The staff make timely adjustments to provide accurate technical data reference, thereby helping to improve the training effect and improve the performance of the competition. The server is a huge comprehensive database of sports information that receives daily training data, competition data, and other types of data compiled by the coaches transmitted from the mobile terminal, and stores and maintains this information. Under this platform, an efficient and fast information management system can greatly solve the problem of in-depth data mining and provide more scientific and effective data analysis results.

The realization of the communication function is that the mobile terminal transfers the data information to the JavaBean object, performs data transmission after serialization, packaging, compression, etc., and then converts it into the corresponding data type through the reverse operation, and finally transmits it to the server-side database.

Taking the on-site monitoring and statistics module of sports training as an example, according to the Apriori algorithm, analyzing the input athletes' daily training data can obtain attribute sets with the same result characteristics among different athletes. This can be used as a starting point for studying problems in training. It is used by coaches as a data reference to facilitate timely adjustment of training plans and competition strategies and is a more accurate and comprehensive basis for decision-making.

## 5. Conclusion

With the continuous advancement of modern technology, people's increasing demand for mobile devices has led to a rapid increase in service requests from mobile devices to edge servers, forcing the reform and innovation of traditional cloud computing. Mobile edge computing technology deploys network servers in various regions, focusing more on users rather than cloud computing, reducing the response time delay of user service requests, thereby improving user service experience and reducing network transmission burden. The energy consumption of each node device is mainly related to the data signal and its transmission path through the node device. The optimal way of controlling the message structure and the reduction of the number of control messages have a very important impact on the energy reduction. Therefore, the CSMA/CA handshake mechanism used in the current traditional wireless sensor network has been optimized in the following three points: first, to effectively control the reduction of the number of messages, and at the same time, the invalid energy consumed in data transmission uses the early response of a node and its adjacent next node to simulate the response of this node to the source node. According to the structural characteristics of traditional wireless sensor networks, optimize the design of the MAC layer to make it more concise and convenient, reduce the number of messages, and thus reduce the energy consumption in the wireless sensor network. Enhance the connection between the nodes in the sensor network to avoid the paralysis of a certain node in the entire network, which will lead to the silence of the entire wireless sensor network. In order to meet the service requests of mobile devices to provide a wide variety of service responses with low latency, this article is based on the mobile edge network computing technology service framework, using SDN's edge network service model and optimization schemes in various situations. Optimize solutions for mobile user service requests and server responses in various environments. It also emphatically introduces the concept of Apriori's multidirectional and highly connected algorithm and its optimization algorithm and then selects the most suitable optimization algorithm under the current environment through multidirectional comparison. The collected data are entered into the database, and then the entire algorithm is optimized and applied to the athlete's daily routine. This article implements simulation experiments based on the Apriori algorithm, analyzes the athletes' daily training data, and provides the coaching staff with scientific and effective data basis, so as to formulate a more suitable personalized exercise program for the athletes, and to enable the coach to have a clearer understanding of the athletes.

## Figures and Tables

**Table 1:** Comparison of five aspects of wireless sensor networks and the Internet of Things.

	Wireless communication	Internet of Things
Definition	“Things” specifically refer to sensors	“Things” include almost all things that can be connected to the internet
Use	Mainly used for information retrieval, generally without feedback operation	Mutual assistance between things and network owners can carry out more complex feedback activities
Information sources		Any device and equipment that can collect information
Basic network	No	Internet, telecommunication network, mobile network, sensor network, etc.
	Thing-to-thing	Object to object, person to person

**Table 2:** Comparison of computational complexity of edge server optimized deployment algorithms.

Algorithm	Computational complexity
EOESPA	*O*(*k*^2^ · |*V*| · *C*_|*V*|_^*k*^)
RNOESPA	*O*(|*V*|^2^ · |*E*| · |*V*|)
KXICA	*O*(*k*^3^ · |*V*|^2^ · *t*)
RESPA	O(1)

**Table 3:** Experimental simulation parameter table.

Experimental parameters	Numerical value
Number of edge servers	[1, 4]
Number of APs	[10, 40]
Number of mobile devices	[200, 1000]
Average data volume of service requests	[20, 100]KB
AP failure probability	[0.05, 0.08]
Link failure probability	[0.02, 0.08]
Network attachment rate	2
AP maximum data transmission rate	1.0 Gbps

**Table 4:** Comparison of computational complexity of optimized deployment algorithms.

Algorithm	Computational complexity
OESDA	0(|*M*| · |*V*|^2^ · (*C*_|*V*|_^*k*^)^*lMl*^)
LAHSDA	*O*(|*M*| · *M* · *k* · (|*V*|+|*M*|))
CEHSDA	0(*k* · |*V*| · |*M*|^2^ · (|*U*|+|*M*|))
SEHSDA	0(*k* · |*M*|^2^ · |*V*|^3^)
GSDA	0(*k* · |*M*| · |*V*|^2^)

**Table 5:** Network topology settings.

Topology	Number of nodes	Number of links
Spiraliglit	15	16
Sago	18	17
Noel	19	25
Shentel	28	35
Missouri	67	83

## Data Availability

The data used to support the findings of this study are available from the corresponding author upon request.
